# Foot lesions in lame cows on 10 dairy farms in Ireland

**DOI:** 10.1186/s13620-015-0039-0

**Published:** 2015-06-07

**Authors:** Joris Somers, Luke O’Grady

**Affiliations:** School of Veterinary Medicine, University College Dublin, Belfield, Dublin 4 Ireland

**Keywords:** Lameness, Foot lesions, Dairy cows, Foot trimming, Grazing, Pasture

## Abstract

**Background:**

Little is known about foot lesions in dairy cattle in Ireland, managed under a pasture based system with housing during the winter and grazing for the rest of the year. Ten Irish dairy herds, with a lameness prevalence ranging from 9 to 17 % were locomotion scored routinely during the 2013 grazing season. Lame cows were foot trimmed and foot lesions recorded.

**Findings:**

11.8 % and 89.6 % of cows had lesions recorded on front and hind feet respectively. No lesions were found in 6.9 % of cows. Sole haemorrhage and white line disease were the most prevalent lesions, and overall 76.8 % of lesions affecting the claw horn were diagnosed on the lateral hind claw.

**Conclusions:**

Treatment success, as measured by improved LS post treatment, was not significantly affected by the LS prior to foot trimming, the presence of lesions or the type of lesion identified. Exposure to both risk factors for lameness at housing and pasture may have resulted in the development of a combination of foot lesions typically associated with zero-grazing or all-year-round grazing management systems.

## Findings

Lameness has a major effect on productivity [1] and compromises welfare in dairy cattle [2]. Prevention, early detection and treatment of lameness is therefore important to reduce these negative effects of lameness on dairy cows [3, 4]. There is a perception that lameness is less of a problem for cows managed at pasture compared to zero-grazing herds [5]. Access to pasture has been reported to reduce the level of lameness and the risk of claw disorders compared to systems with limited outdoor access [6–9]. The annual incidence of lameness and the lesions developed are similar in both grazing and housed production systems. [5] Grazing cows however, are at greater risk of developing white line disease, especially those cows grazing during the day and housed at night. [10] Irish dairy cattle are exposed to risk factors for lameness associated with housing and grazing [11]. Most international studies on dairy cow foot lesions involve either cattle housed the majority of the time or cattle kept outdoors all year round. Little is known about foot lesions in cattle managed under a pasture based system with housing only during part of the winter and grazing for the rest of the year, typical of the management in Irish dairy herds. The objectives of this descriptive study were to 1) identify the type of foot lesions found in lame dairy cows managed under a pasture based production system in Ireland, 2) describe the distribution of foot lesions between front and hind limb and lateral and medial claw and 3) describe the change in locomotion score (LS) following treatment based on follow-up lameness assessment.

This investigation was conducted as part of a larger study on lameness and fertility, carried out on 10 dairy farms (Table 1) in Co. Kildare, Ireland. These 10 farms participated in an on-going herd health programme, conducted by University College Dublin. Dairy cow lameness was routinely monitored as part of the programme. Four routine lameness scoring visits to each study farm were conducted during the 2013 grazing season at an average interval of 45 days. As the cows were leaving the milking parlour after morning milking, the entire milking herd was visually assessed for lameness by the first author using a 5-point LS scale [12]. Using this scale, each cow was awarded LS from 1 to 5 based on the presence or absence of an arched back when standing and walking and the observation of gait abnormalities. Lame cows were defined as having LS of 3, 4 or 5. Between 4th April and 31st May, cows were presented for treatment to one and the same professional foot trimmer within 8 days of being diagnosed as lame. This foot trimmer was provided by Farm Relief Services (Roscrea, Co. Tipperary) and trained in the Dutch foot trimming method. Additional treatment in conjunction with corrective foot trimming consisted of rubber or wooden blocks placed on healthy claws to relieve affected claws whenever this was indicated. Cow identification, date of treatment and lesions found were recorded by the first author using the DairyCo. (Glocestershire, United Kingdom) hoofcare recording chart. A photograph of the affected foot was also taken at this time. All 4 feet were examined and findings recorded. The list of lesions comprised of foot abnormalities associated with lameness, although not all may be considered painful, for example long toe. Lesions were classified, based on their potential aetiology, as either infectious or non-infectious causes of lameness and the definition of the foot lesions is presented in Table 2 [13, 14]. Lesions classified as infectious causes of lameness were digital dermatitis (DD), interdigital necrobacillosis (IN), interdigital dermatitis (ID), and heel horn erosions (HHE). Claw disorders classified as non-infectious causes of lameness were sole haemorrhage (SH), sole ulcer (SU), white line disease (WLD), double sole (DS), toe ulcer (TU), axial wall fissure (AF), vertical wall fissure (VF), horizontal wall fissure (HF) and interdigital hyperplasia (IH). Long toe (LT) was also included in this category as a foot trimming finding associated with lameness. The affected claw, lateral or medial, was also recorded for non-infectious lesions associated with the claw horn. Treatment outcome was based on the results of the routine lameness scoring visit following foot trimming. The average number of days between treatment and the following locomotion scoring session was 38.9 days (SD: ±6.9 days). If animals were awarded LS < 3 during this locomotion scoring session, treatment outcome was considered successful. All data were transcribed to Excel and descriptive analysis was performed on farm information and lesion type and lesion location data. Cross tabulation was used to describe the proportion of lesion type and the number of lesions per cow for parity and the different levels of lameness. Stata (StataCorp, Texas, USA) was used to perform Chi-square and One-way Anova analysis of the data.

**Table 1:** General information about the 10 study farms, number of milking cows present during the study, % lame cows (LS ≥ 3), number of cows submitted for treatment by foot trimming, number of lesions recorded during foot trimming and % of lesions categorised as infectious or non-infectious

Farm	1	2	3	4	5	6	7	8	9	10
No. of cows	146	115	185	100	80	150	72	120	157	79
% LS ≥ 3	8.9	9.6	16.9	12.8	8.8	13.3	13.8	9.2	17.2	13.9
No. treated	11	11	26	10	7	20	10	11	27	11
No. of lesions recorded	19	44	74	44	24	78	27	35	105	39
% infectious lesions	10.5	9.1	10.8	38.6	8.3	7.7	11.1	17.1	20.9	10.3
% non-infectious lesions	89.5	90.9	89.2	61.4	91.7	92.3	88.9	82.9	79.1	89.7

**Table 2:** Definitions of foot lesions as recorded during foot trimming of 144 dairy cows

Digital dermatitis	Proliferative inflammation of the dorsal or plantar – palmar skin. Commonly found adjacent to the heels or less commonly in the proximal part of the interdigital space
Interdigital necrobacillosis	Acute necrotising inflammation of the interdigital skin and underlying tissues, with swelling above the coronary band and in the interdigital space
Interdigital dermatitis	Inflammation of the interdigital skin without extension to deeper tissues
Heel horn erosions	Pits and pockmarks, with parallel horizontal grooves on the bulb of the heel. Sometimes the horn is separated forming flaps
Sole haemorrhage	Haemorrhagic discoloration covering ≥ 50 % of the sole or a smaller area with deep intense colour
Sole ulcer	Circumscribed loss of the sole horn exposing the corium of the solar surface, located in the region of the sole-bulb junction, usually nearer the axial margin with or without additional affection of deeper structures of the claw
White line disease	Separation of the abaxial sole from the wall; disrupted continuity of white line with the presence of necrosis, organic material or an abscess
Double sole	Under run of the sole horn, two soles separated by a space
Toe ulcer	Focal area of necrosis or an abscess located at the tip of the claw, undermining the sole horn. Severe cases can be associated with distal phalangeal osteitis
Axial wall fissure	Deep fissure on the axial surface of the claw, parallel to the dorsal wall
Vertical wall fissure	Vertical fissure of the wall horn, usually located on the dorsal or dorso-lateral aspect of the claw
Horizontal wall fissure	Fissure of the wall horn, parallel to the coronary band
Long toe	Length of the dorsal aspect of the claw ≥ 8 cm and angle of the dorsal wall of the claw < 50°
Interdigital hyperplasia	Fibrous proliferation of the interdigital skin causing a mass that protrudes between the claws; which could be inflamed or not

Adapted from Greenough [13] and Tadich et al. [14]

The overall prevalence of lameness in the group of farms was 12 %. Across the ten farms, 144 lame cows were presented for foot trimming. Lesions were identified in 134 cows (93.1 %), lesions were absent in 10 cows (6.9 %). Infectious type lesions were diagnosed in 50 cows (34.7 %), 129 cows (89.6 %) had at least 1 non-infectious type lesion. The number of cows diagnosed with each type of lesion is shown in Table 3. Five cows (3.5 %) were diagnosed with infectious lesions only, 45 cows (31.3 %) with both infectious and non-infectious lesions and 84 cows (58.3 %) had non-infectious lesions only. The number of cows receiving treatment for lameness and the proportion of infectious and non-infectious lesions found per farm is detailed in Table 1. Both categories of lesions were recorded on all 10 farms, but 3 of the farms had a higher prevalence of infectious lesions. DD was present in cows on 8 out of 10 farms, HHE was found in 50 % of the herds. SH and WLD were diagnosed in cows from all herds.

**Table 3:** The number of cows diagnosed with each lesion and the total number of lesions (*n* = 489) found during foot trimming of 144 lame cows on 10 dairy farms in Ireland

	Infectious lesions				Non-infectious lesions									
	DD	IN	ID	HHE	SH	SU	WLD	DS	TU	AF	VF	HF	LT	IH
Cows	40	0	0	15	91	18	75	33	9	9	2	0	21	12
Lesions	53	0	0	21	145	20	115	45	10	9	2	0	54	15

A total of 489 lesions were identified, the number of lesions recorded per cow ranged from 0 to 9 (median 3). Seventy four (15.1 %) infectious lesions and 415 (84.9 %) non-infectious lesions were recorded. SH and WLD made up the majority of lesions, respectively accounting for 29.7 % and 23.5 % of all lesions encountered. Table 4 shows to which extent a lesion was diagnosed in a cow in combination with another lesion type. Overall, 64.2 % and 52.2 % of cows were diagnosed with a lesion in combination with SH or WLD respectively. A single lesion was found in 6.4 % of lame cows. The mean number of lesions found per cow for 3 lactation groups is shown in Table 5. The feet of 10 (6.9 %) 1st lactation, 11 (7.6 %) 2nd lactation and 123 (85.4 %) ≥ 3rd lactation cows were examined. A significantly higher mean number of TU per cow was found in the 2nd lactation group compared to the ≥ 3rd lactation group. The mean number of LT per cow was also significantly higher for the 2nd lactation group compared to the other two groups. Increasing parity was associated with an increasing trend in mean number of SH diagnosed per cow, however this was not significant (p = 0.08).

**Table 4:** The proportion of cows, expressed as %, diagnosed with a combination of 2 different lesions or with a single (Single) lesion type

Lesion	No.	DD	HHE	SH	SU	WLD	AF	TU	IH	VF	DS	LT	Single
DD	40	-	12.5	67.5	7.5	55.0	2.5	2.5	10.0	2.5	32.5	5.0	5.0
HHE	15	33.3	-	53.3	13.3	53.3	6.7	0	13.3	0	13.3	13.3	0
SH	91	29.7	8.8	-	13.2	56.0	6.6	6.6	9.9	1.1	24.2	14.3	14.3
SU	18	16.7	11.1	66.7	-	55.6	5.6	11.1	22.2	0	50.0	16.7	5.6
WLD	75	29.3	10.7	68.0	13.3	-	4.0	5.3	9.3	1.3	32.0	12.0	12.0
AF	9	11.1	11.1	66.7	11.1	33.3	-	0	22.2	0	22.2	55.6	0
TU	9	11.1	0	66.7	22.2	44.4	0	-	0	11.1	44.4	11.1	11.1
IH	12	33.3	16.7	75.0	33.3	58.3	16.7	0	-	0	41.7	8.3	8.3
VF	2	50.0	0	50.0	0	50.0	0	50.0	0	-	0	0	0
DS	33	39.4	6.1	66.7	27.3	72.7	6.1	12.1	15.2	0	-	9.1	0
LT	21	9.5	9.5	61.9	14.3	42.9	23.8	4.8	4.8	0	14.3	-	14.3

**Table 5:** The mean number of lesions found per cow across 3 lactation groups

	1st Lactation (*n* = 10)	2nd Lactation (*n* = 11)	≥3rd Lactation (*n* = 123)	One-way Anova *p*-value
Infectious	0.50	0	0.56	0.11
Non-infectious	1.80	3.55	2.90	0.13
Total	2.30	3.64	3.47	0.21
DD	0.40	0	0.40	0.14
HHE	0.10	0	0.16	0.50
SH	0.50	0.73	1.07	0.08
SU	0.10	0.18	0.14	0.89
WLD	0.80	0.64	0.81	0.84
AF	0	0	0.07	0.45
TU	0.10^a,b^	0.27^b^	0.05^a^	0.04
IH	0	0.09	0.11	0.64
VF	0	0	0.02	0.88
DS	0.30	0.55	0.29	0.46
LT	0^a^	1.09^b^	0.34^a^	0.03

First and second lactation animals were analysed in separate groups. Third and higher (≥3) lactation animals were combined into 1 group. ^a^ and ^b^ indicate a significant (*p* < 0.05) difference in the mean number of lesions found per cow for that specific lesion between the different lactation groups

The distribution of lesions between the front and hind limbs and the lateral and medial claws is shown in Table 6. A significant higher proportion of cows (*p* < 0.05) was diagnosed with lesions on the hind feet. Lesions were present on the front and hind limbs of 17 and 129 cows (11.8 % and 89.6 %) respectively. Five cows (3.5 %) were diagnosed with lesions on the front limbs only, 12 (8.3 %) cows had lesions on both front and hind limbs whilst 117 (81.3 %) cows had only hind limb lesions. In the front limb, non-infectious lesions affecting the claw horn were found on a lateral claw only in 1 cow (6.2 %), a medial claw only in 13 cows (81.3 %) and on both claws in 2 cows (12.5 %). In the hind limb, 77 cows (63.1 %) had lesions on a lateral claw only, 3 cows (2.5 %) had lesions on a medial claw only and 42 cows (34.4 %) had lesions on both claws. Evaluation of the distribution of lesions between front and hind feet found significantly more lesions (*p* < 0.05) on the hind feet. Thirty lesions (6.1 %) were found on the front limb, 459 lesions (93.9 %) were recorded for the hind limbs. Of the lesions that could be allocated to a specific claw, 4 and 22 were found on a front lateral and medial claw respectively (15.4 % and 84.6 % respectively). The hind lateral and medial claws were affected by 308 and 67 lesions respectively (82.1 % and 17.9 % respectively).

**Table 6:** The number of cows diagnosed with lesions to the front or hind limb and the proportion of cows with lateral (L) and medial (M) claw horn lesions only, both lateral and medial (L + M) claw horn lesions or lesions not affecting the claw horn (Other) of the respective limbs. And the number of lesions allocated to the front and hind limb and the proportion of lesions affecting the lateral (L) and medial (M) claw horn or lesions not affecting the claw horn (Other) of the respective limbs

		Cow	Lesions
Front limb	No.	17	30
%	L only	5.9	13.3
	M only	76.5	73.4
	L + M	11.8	-
	Other	5.9	13.3
Hind limb	No.	129	459
%	L only	59.7	67.9
	M only	2.3	14.6
	L + M	32.6	-
	Other	5.4	18.3

Increased severity of lameness prior to treatment (Table 7), defined as LS 4 vs LS 3, was not influenced by the proportion of cows diagnosed with lack of lesions, infectious lesions only, non-infectious lesions only or both infectious and non-infectious lesions for both levels of lameness. The lesion profile however did show a significant (*p* = 0.01) difference between LS 4 and LS 3 cows. This difference was numerically driven by the higher proportion of SH and LT in LS 3 cows and the higher proportion of SU, WLD, AF, TU and VF in LS 4 cows.

**Table 7:** Chi-square tests to compare the proportions of the number of cows diagnosed with lack of lesions, infectious lesions only, non-infectious lesions only or both infectious and non-infectious lesions and the number of specific lesions found during foot trimming in relation to the locomotion score (LS) recorded for each cow prior to foot trimming

		% LS 3 (*n* = 95)		% LS 4 (*n* = 49)		Fisher’s Exact Test
	Total	No.	%	No.	%	*p*-value
No lesions found	10	9	9.5 s	1	2.0	0.32
Infectious only	5	4	4.2	1	2.0	
Non-infectious only	84	55	57.9	29	59.2	
Both	45	27	28.4	18	36.7	
DD	53	32	10.5	21	11.5	
HHE	21	15	4.9	6	3.3	
SH	145	100	32.8	45	24.6	
SU	20	10	3.3	10	5.5	
WLD	115	69	22.6	46	25.1	
AF	9	3	1.0	6	3.3	0.01
TU	10	3	1.0	7	3.8	
IH	15	12	3.9	3	1.6	
VF	2	0	0	2	1.1	
DS	45	24	7.9	21	11.5	
LT	54	38	12.5	16	8.7	

One hundred forty cows received a routine LS following treatment (Table 8). Treatment success was not significantly (*p* = 0.22) different for cows with LS 3 vs LS 4 prior to foot trimming, however a numerical difference of 58.8 % vs 50.0 % respectively was noted. Differences in treatment success were not affected by the presence of lesions or the type of lesion identified during foot trimming. Overall a total of 78 cows (55.7 %) were recorded as cured during the follow-up locomotion scoring session.

**Table 8:** Success of treatment based on the first routine locomotion scoring session following foot trimming. 140 of the cows that were treated were submitted for follow-up LS. 78 cows received LS < LS 3, resulting in an overall success of treatment of 55.7 %

	No.	Cured	
		No.	%
Overall	140	78	55.7
Prior LS 3	94	55	58.8
Prior LS 4	46	23	50.0
Lack of lesions	10	6	60.0
Lesion present	130	72	55.4
Infectious only	5	3	60.0
Non-infectious only	81	43	53.1
Both	44	26	59.1

This study found foot lesions in lame dairy cattle associated with housing environment and grazing. Most international studies [1, 3, 15, 16] were based on whole herd foot trimming records, whereas the present study only included lame cows. Due to time and financial constraints it was not possible to include LS 1 or LS 2 cattle in the study. As not every claw disorder is associated with lameness, the lesion profile found based on lame cows only may be different from that found during routine whole herd foot trimming. Olmos et al. [5] concluded that cows require 85 days at pasture to recover from the risk factors for lameness related to housing and the post-partum period. Typically, Irish dairy cattle are housed during part of the winter and the minimum of 85 days at pasture was not met for all the cows in the present study. Exposure to both risk factors for lameness at housing and pasture [11, 17] may explain a similar distribution of lesions found in confinement housed dairy herds. Levels of SH and SU found in housed dairy cattle, are similar to those found in the present study, however levels of infectious lesions are higher and WLD is found less frequently [3, 16, 18]. Grazing has been identified as a risk factor for WLD in both full time and part time grazing herds [10]. In New Zealand grazing dairy cows, WLD is by far the most common foot lesion and multiple foot lesions on the same foot or infectious lesions are absent or rare [1]. Both SH and HHE are not associated with increasing LS, while WLD is commonly diagnosed as a cause of lameness [16, 19, 20]. Comparing the lesion profile between LS 3 and LS 4 cows in the present study, it can be concluded that SU, WLD, AF, TU and VF elicit a higher pain response compared to other foot abnormalities found during foot trimming of lame cattle. Higher proportions of both infectious and non-infectious foot lesions were found in lame cows kept at pasture, compared to housed lame cows, but the severity of lesions was higher in housed lame cows [5, 10]. Full time grazing poses an increased risk for the development of SU, WLD and DD in dairy cows [10]. However, Somers et al. [7] found a higher number of claw disorders in zero-grazing cows compared to grazing cows. Part time grazing of cows tends to decrease the risk of DD and SU and increase the risk of WLD compared to confinement housed dairy cattle [10]. Holzhauer et al. [8] reported a lower prevalence of DD, ID and HHE in grazing herds during the grazing season but not a lower prevalence of SH compared to zero-grazing herds. Interestingly, ID and IN were not diagnosed in any of the feet examined in the present study. Both these conditions are associated with unhygienic underfoot conditions that lead to prolonged exposure of the interdigital skin to slurry [13]. Dairy cows at pasture typically are less exposed to such conditions compared to housed cattle. Another possibility is that due to the presence of multiple lesions per cow, small ID lesions were not diagnosed. HF was also not diagnosed in the present study. This lesion is often associated with nutritional or metabolic stress and only a cause of lameness in severe presentations [13].

In a study from south Chile, where dairy cows are managed in a similar way compared to Ireland, cattle had an average of 3 foot lesions [16]. This is the same number as found in the present study. Only 6.5 % of cows were diagnosed with a single type of lesion and the majority were diagnosed with a lesion in combination with SH or WLD. As a result, it is difficult to assess which is the primary lameness causing lesion in a cow. A large proportion of cows diagnosed with AF were also diagnosed with LT. AF is widespread among grazing cows that walk long distances on gravel tracks and is more common among dairy cows with shallow dorsal wall angle and claw imbalance, as seen with LT [21]. Overall, 76.8 % of lesions affecting the claw horn were diagnosed on the lateral hind claw. This finding is similar to that seen in a UK study based on trimming records involving more than 20,000 cows [22].

The objective of this study was to provide an observational analysis of foot trimming records of a relatively small number of lame dairy cows. As only lame cattle were examined, associations between lesions recorded and LS or risk factors for lesions could not be made. Future work should include foot trimming results from non-lame cows and follow-up lesion identification to fully understand the dynamics of foot lesions in Irish dairy cows.

## Abbreviations

LS: Locomotion score
DD: Digital dermatitis
IN: Interdigital necrobacillosis
ID: Interdigital dermatitis
HHE: Heel horn erosions
SH: Sole haemorrhage
SU: Sole ulcer
WLD: White line disease
DS: Double sole
TU: Toe ulcer
AF: Axial wall fissure
VF: Vertical wall fissure
HF: Horizontal wall fissure
LT: Long toe
IH: Interdigital hyperplasia
